# Aluminum Fluoride-18 Labeled Mannosylated Dextran: Radiosynthesis and Initial Preclinical Positron Emission Tomography Studies

**DOI:** 10.1007/s11307-023-01816-7

**Published:** 2023-04-04

**Authors:** Putri Andriana, Konstantina Makrypidi, Heidi Liljenbäck, Johan Rajander, Antti Saraste, Ioannis Pirmettis, Anne Roivainen, Xiang-Guo Li

**Affiliations:** 1 Turku PET Centre, University of Turku, Kiinamyllynkatu 4-8, FI-20520 Turku, Finland; 2 Institute of Nuclear and Radiological Science and Technology, Energy and Safety, NCSR “Demokritos”, 15310 Athens, Greece; 3 Turku Center for Disease Modeling, University of Turku, FI-20520 Turku, Finland; 4 Accelerator Laboratory, Åbo Akademi University, FI-20520 Turku, Finland; 5 Turku PET Centre, Turku University Hospital, FI-20520 Turku, Finland; 6 Heart Center, Turku University Hospital and University of Turku, FI-20520 Turku, Finland; 7 InFLAMES Research Flagship Center, University of Turku, FI-20520 Turku, Finland; 8 Department of Chemistry, University of Turku, FI-20014 Turku, Finland

**Keywords:** Fluorine-18, Dextran, Mannose, Mannose receptor, PET

## Abstract

**Purpose:**

In addition to being expressed on liver sinusoidal endothelial cells, mannose receptors are also found on antigen-presenting cells, including macrophages, which are mainly involved in the inflammation process. Dextran derivatives of various sizes containing cysteine and mannose moieties have previously been labeled with ^99m^Tc and used for single-photon emission computed tomography imaging of sentinel lymph nodes. In this study, we radiolabeled 21.3-kDa D10CM with positron-emitting ^18^F for initial positron emission tomography (PET) studies in rats.

**Procedures:**

D10CM was conjugated with 1,4,7-triazacyclononane-1,4,7-triacetic acid (NOTA) chelator and radiolabeled with the aluminum fluoride-18 method. The whole-body distribution kinetics and stability of the intravenously administered tracer were studied in healthy male Sprague-Dawley rats by *in vivo* PET/CT imaging, *ex vivo* gamma counting, and high-performance liquid chromatography analysis.

**Results:**

Al[^18^F]F-NOTA-D10CM was obtained with a radiochemical purity of >99% and molar activity of 9.9 GBq/μmol. At 60 minutes after injection, an average of 84% of the intact tracer was found in the blood, indicating excellent *in vivo* stability. The highest radioactivity concentration was seen in the liver, spleen, and bone marrow, in which mannose receptors are highly expressed under physiological conditions. The uptake specificity was confirmed with *in vivo* blocking experiments.

**Conclusions:**

Our results imply that Al[^18^F]F-NOTA-D10CM is a suitable tracer for PET imaging. Further studies in disease models with mannose receptor CD206-positive macrophages are warranted to clarify the tracer’s potential for imaging of inflammation.

**Graphical abstract:**

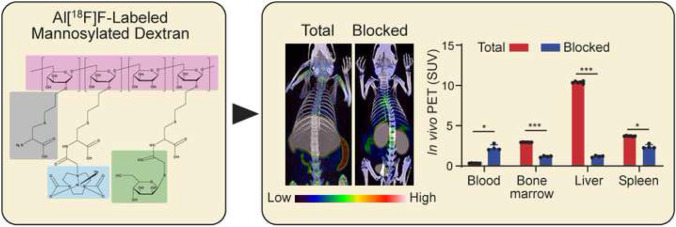

**Supplementary Information:**

The online version contains supplementary material available at 10.1007/s11307-023-01816-7.

## Introduction

New carbohydrate-derived radiopharmaceuticals have recently reached major clinical breakthroughs. For example, ^99m^Tc-Tilmanocept (Lymphoseek®) has been approved for clinical use by the U.S. Food and Drug Administration and the European Medicines Agency. ^99m^Tc-Tilmanocept (17 kDa) is a mannosylated dextran developed for imaging of sentinel lymph nodes with single-photon emission computed tomography [1]. Indeed, dextran is a platform molecule that has been used in many types of pharmaceutical applications, and mannose is useful for decoration of drug molecules to achieve specific targeting. The attachment of mannose moieties to the dextran backbone may direct the radiotracer to the carbohydrate-recognition domain of C-type lectin mannose receptor (cluster of differentiation 206, CD206) on the surface of macrophages and dendritic cells. A study by Núñez and coworkers showed that uptake of mannosylated dextran in lymph nodes was four times higher than uptake of corresponding radiolabeled dextran without mannosylation [2]. In a recent report, Pirmettis and coworkers labeled a mannosylated dextran with ^99m^Tc(CO)_3_ using a cysteine moiety as a chelator. The generated compound, ^99m^Tc(CO)_3_-DCM20, has been studied for the detection of sentinel lymph nodes in preclinical settings, and promising results have been obtained on single-photon emission computed tomography [3]. Our research focuses on the development of radiopharmaceuticals for positron emission tomography (PET) imaging, and we have radiolabeled many types of molecules, including oligosaccharides for mannose receptor targeting [4]. In this study, the aluminum fluoride-18 (Al[^18^F]F) labeling technique was used to radiosynthesize a 21.3 kDa dextran containing cysteine and mannose moieties (D10CM), which we refer to as Al[^18^F]F-NOTA-D10CM, where NOTA denotes the chelator 1,4,7-triazacyclononane-1,4,7-triacetic acid used for attaching Al[^18^F]F to the structure. In addition, we report a preclinical evaluation study of Al[^18^F]F-NOTA-D10CM in healthy rats.

## Materials and Methods

### Materials

The mannosylated and cysteinylated dextran derivative D10CM (21.3 kDa) was synthesized with a method similar to that previously described [3, 5, 6]. The compound 2,2′-(7-(2-((2,5-dioxopyrrolidin-1-yl)oxy)-2-oxoethyl)-1,4,7-triazonane-1,4-diyl) diacetic acid (NOTA-NHS, *N*-hydroxysuccinimide-activated ester of NOTA) was purchased from CheMatech (Dijon, France). All other chemicals were purchased from commercial sources.

### Preparation of NOTA-D10CM

NOTA-NHS ester (117 mg) dissolved in 2 mL of dimethyl sulfoxide was added to a solution of D10CM (100 mg) in 4 mL of borate buffer (0.1 M, pH 9.0). The reaction mixture was stirred at room temperature for 18 hours and then transferred to an ultrafiltration cell (model 8050, Millipore Corp, Bedford, MA) fitted with an ultrafiltration membrane (YM03). The volume was adjusted to 50 mL with deionized water, and the solution was then concentrated to 5 mL by applying nitrogen gas under pressure directly to the ultrafiltration cell. The procedure was repeated four times, and the residue was finally lyophilized to give the product as a white solid (100 mg).

### Preparation of AlCl_3_ Stock Solution in Acetate Buffer

The stock solution of AlCl_3_ (2 mM) was prepared in acetate buffer (pH 4.0, 1 M). The acetate buffer was prepared by dissolving sodium acetate (8.2 g, 100 mmol) in TraceSELECT water. The pH was carefully adjusted to 4.0 with acetic acid and magnetic stirring, and the final volume was adjusted to 100 mL. Aluminum chloride hydrate (AlCl_3_ 6.5H_2_O, 50.1 mg, 200 μmol) was added to the acetate buffer. The stock solution was divided into aliquots and stored at 4°C.

### Radiosynthesis of Al[^18^F]F-NOTA-D10CM

Radiosynthesis was performed with a custom-made remote-controlled device (DM Automation, Nykvarn, Sweden), as previously reported [7]. [^18^F]Fluoride was produced in an ^18^O(p,n)^18^F nuclear reaction with a cyclotron using H_2_[^18^O]O as the target and extracted into an anion-exchange cartridge (Chromafix 30 PS-HCO_3_^−^, Macherey-Nagel, Düren, Germany; preconditioned with 1 mL of ethanol followed by 1 mL of water). Subsequently, physiological saline (9 mg/mL, 220 μL) was used to elute [^18^F]fluoride into the reaction vial prefilled with NOTA-D10CM (1.5 mg) in 50 μL TraceSELECT water (Honeywell, Morristown, NJ), ascorbic acid (150 mM, 40 μL), AlCl_3_ (2 mM) in sodium acetate buffer (pH 4.0, 1 M, 25 μL), and acetonitrile (50 mM, 60 μL). The mixture was heated at 100°C for 13 minutes and cooled down to 40°C before addition of water (810 μL) containing 0.1% trifluoroacetic acid (TFA) and ascorbic acid in water (150 mM, 90 μL). The product was purified using high-performance liquid chromatography (HPLC) equipped with a semipreparative C18 Jupiter Proteo column (250 × 10 mm, 4 μm, 90 Å; Phenomenex, Torrance, CA) with mobile phases of water containing 0.1% TFA (solvent A) and acetonitrile containing 0.1% TFA (solvent B). From 0 to 1 minute, the flow rate was increased from 1 to 4 mL/minute with 5% solvent B and 95% solvent A. The solvent B content was increased to 30% over 1–20 minutes, and the fraction of Al[^18^F]F-NOTA-D10CM was collected to a vial prefilled with water (30 mL) and ascorbic acid (150 mM, 400 μL). The product was extracted into a preconditioned (5 mL of ethanol followed by 10 mL of water) tC18 cartridge (Waters, Milford, CT), which was subsequently rinsed with 5 mL of water. Then, the final product was eluted out from the tC18 cartridge with 1 mL of 30% ethanol (300 μL ethanol, 100 μL 150 mM ascorbic acid in water, and 600 μL water) into the end product bottle, which was prefilled with 15 mM ascorbic acid in saline (1.5 mL), and 0.1 M phosphate-buffered saline (PBS) was then used to adjust the pH to make it suitable for animal studies.

### Quality Analysis of Al[^18^F]F-NOTA-D10CM

The radiochemical purity of the end product sample (0.3-0.8 MBq) was determined using an HPLC system (Hitachi; Merck, Darmstadt, Germany) equipped with a Jupiter Proteo column (reversed-phase C18, 150 × 4.6 mm, 300 Å; Phenomenex) and a radioactivity detector (Radiomatic 150TR flow-through, Packard, Meriden, CT). Solvent A was water containing 0.1% TFA, and solvent B was acetonitrile containing 0.1% TFA. In the elution protocol, the content of solvent B was increased from 8% to 40% over 0–9 minutes and then maintained at 40% over 9–10 minutes at a flow rate of 1 mL/minute. The stability of Al[^18^F]F-NOTA-D10CM in the final product formulation buffer was assessed by HPLC analysis of aliquots taken at 1, 2, 3, and 4 hours after the end of synthesis (EOS).

### Log*D* Measurement

PBS (600 μL) and 1-octanol (600 μL) were added to a 1.5 mL Eppendorf tube followed by the addition of 5 kBq of Al[^18^F]F-NOTA-D10CM. The mixture was vortexed for 5 minutes, and then the water and organic phases were separated by centrifugation at 14,100 ×*g* for 3 minutes at room temperature. A 400 μL aliquot was taken from both phases for gamma counting (Wizard 3″, PerkinElmer/Wallac, Turku, Finland). The measurements were performed in triplicate. The Log*D* value was calculated using the formula: $logD={Log}_{10}\left(\frac{counts\ in\ 1- octanol}{counts\ in\ PBS}\right)$.

### Determination of Molar Activity

The molar activity was determined by means of HPLC analysis using a wavelength of 220 nm under UV detection. Five NOTA-D10CM samples at concentrations of 1, 2, 4, 6, and 8 μM were analyzed in triplicate and used to generate a calibration curve. The HPLC method was the same as that described above for quality control. Then, 50 μL samples of radioactivity-decayed final product were analyzed by HPLC, and the concentration (*c*, nmol/mL) of the product was calculated using the calibration curve. Finally, the molar activity at EOS was calculated as follows:

$${A}_m=\frac{Total\ radioactivity\ at\ EOS\ (MBq)}{c\ \left(\frac{nmol}{mL}\right)\times volume\ (mL)\ of\ final\ product\ at\ EOS}$$

### PET Studies

Healthy male Sprague-Dawley rats (*n* = 6, body weight 268.3 ± 31.8 g, age 7–8 weeks) and smaller sized rats of the same strain for the *in vivo* blocking study (*n* = 4, body weight 94.9 ± 3.7 g, age 4 weeks) were obtained from the Central Animal Laboratory, University of Turku, Turku, Finland. The PET/computed tomography (CT) imaging was acquired using an Inveon Multimodality scanner (Siemens Medical Solutions, Knoxville, TN). Rats were anesthetized with isoflurane (4-5% for induction and 1.5-2% for maintenance), and the tail vein was cannulated. A CT scan was first performed for attenuation correction and anatomical reference, and then a 60 minute PET acquisition was started at the time of intravenous injection of Al[^18^F]F-NOTA-D10CM (51.6 ± 1.8 MBq, 431.1 ± 233.4 μL). To perform the blocking experiment, 100-fold molar excess of mannan from *Saccharomyces cerevisiae* (Sigma-Aldrich M7504)) in saline (400 μL) was injected 15 minutes before the Al[^18^F]F-NOTA-D10CM injection. PET data obtained in a list-mode were reconstructed with an ordered subsets expectation maximization 3-dimensional (OSEM-3D) algorithm into 30 × 3 s, 9 × 10 s, 4 × 30 s, 5 × 60 s, and 10 × 300 s time frames.

After the PET/CT imaging, animals were euthanized, and various tissues were collected, weighed, and measured with a gamma counter (Triathler 3″, Hidex, Turku, Finland). The radioactivity of the excised tissues was expressed as a percentage of the injected radioactivity dose per gram of tissue (%ID/g), corrected for the radioactivity remaining in the cannula and tail.

PET/CT image analysis was performed using Carimas 2.10 software (Turku PET Centre, Turku, Finland, www.turkupetcentre.fi/carimas/). Regions of interest (ROIs) were manually defined in main organs using CT for anatomical reference. Time-activity curves were extracted from the 60 minute PET data and expressed as standardized uptake value (SUV) versus time after injection.

All animal experiments were approved by the national Project Authorization Board in Finland (license number ESAVI/43134/2019) and carried out in compliance with the EU Directive 2010/EU/63 on the protection of animals used for scientific purposes.

### *In Vivo* Stability of Al[^18^F]F-NOTA-D10CM

Healthy male Sprague-Dawley rats (*n* = 6, body weight 457.5 ± 15.5 g, age 15 weeks) were intravenously injected with Al[^18^F]F-NOTA-D10CM (dose 49.1 ± 2.1 MBq), and blood samples were collected into heparinized tubes at 5, 15, 30, 45 and 60 minutes (*n* = 3-4 for each time point) postinjection, using the tail-cut method. Plasma and cells were separated by centrifugation (5 minutes at 14,000 ×*g* at 4°C) and gamma counted (3″ NaI system, Triathler, Hidex Oy, Turku, Finland). Plasma proteins were precipitated with 10% sulfosalicylic acid and pelleted by centrifugation (2 minutes at 14,000 ×*g* at room temperature). The radioactivity concentration of the supernatant and the pellet were determined by gamma counting. The plasma supernatant was filtered through a 0.45 μm Minispike filter (Waters Corporation, Milford, MA), diluted with 0.1% TFA in water to 1 mL, and analyzed by radio-HPLC with a C18 Jupiter Proteo semipreparative column (Phenomenex, 250 × 10 mm, 5 μm, 90 Å) and conditions of 0.1% TFA in water (solvent A) and 0.1% TFA in acetonitrile (solvent B), a gradient of 5% B from 0-3 minutes gradually increasing from 5% to 60% B until 15 minutes, and a 5 mL/minute flow rate.

### Immunohistochemical Staining

Liver, spleen, and bone marrow samples were formalin-fixed, paraffin-embedded, and cut into 4-μm sections. The slides were deparaffinized and rehydrated, followed by antigen retrieval (citrate buffer, pH 6.0, 20 minutes in pressure cooker). After washing (Tris-HCl 0.05 M, pH 7.6 with 0.05% Tween 20) and blocking endogenous peroxidase (3% hydrogen peroxidase), the slides were incubated with the anti-mannose receptor (CD206/MRC1) antibody (ab64693, working dilution 1:10000; Abcam, Cambridge, UK) for 60 minutes at room temperature. After washes, the slides were incubated with BrightVision horseradish peroxidase conjugated goat anti-mouse secondary antibody (DPVR110HRP; WellMed, Duiven, The Netherlands) for 30 minutes at room temperature. After 3,3-diaminobenzidine reaction (BrightDAB, BS04-110; WellMed), the sections were counterstained with Mayer’s hematoxylin, and mounted with Pertex. The slides dried overnight were scanned with a digital slide scanner (Pannoramic P1000, 3DHistech Ltd., Budapest, Hungary).

### Statistical Analysis

Results are expressed as mean ± standard deviation (SD). The significance of differences between the groups was determined with unpaired Student’s *t* tests. *P*-values <0.05 were considered statistically significant.

## Results

### Synthesis of NOTA-D10CM

The mannosylated dextran D10CM was prepared according to the published method [3, 6]. NOTA-D10CM was synthesized in a conjugation reaction between D10CM and the NOTA-NHS in borate buffer at pH 9. The reaction was accomplished over 18 hours at room temperature followed by product purification by ultrafiltration with a 3 kDa cut-off. NOTA-D10CM was obtained as a sound white powder after lyophilization and subjected to analysis with nuclear magnetic resonance. Results revealed that NOTA-D10CM had 7 free and 17 mannosylated S-derivatized cysteines with a minimum of one NOTA chelator for each D10CM molecule (Supplementary Fig. 1).

### Preparation of Al[^18^F]F-NOTA-D10CM

**Fig. 1:**
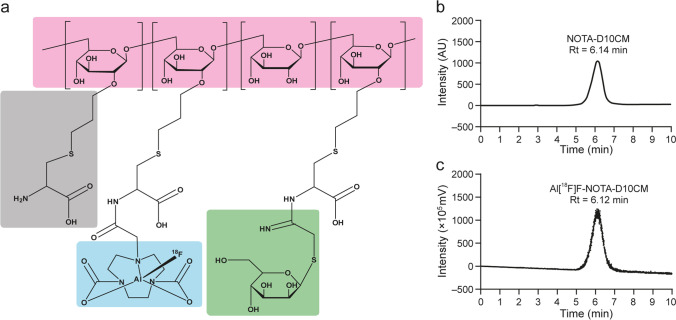
Chemical structure and quality control of Al[^18^F]F-NOTA-D10CM. (**a**) In the structure, the dextran backbone is indicated in pink, cysteine in gray, NOTA chelator in blue, and mannose moiety in green. Representative quality control HPLC chromatographs of (**b**) NOTA-D10CM under UV detection and (**c**) Al[^18^F]F-NOTA-D10CM under radioactivity detection

We produced [^18^F]fluoride in an ^18^O(p,n)^18^F nuclear reaction using H_2_[^18^O]O as a target in the cyclotron. The radiosynthesis was performed with a remote-controlled radiosynthesis device [7]. An anion-exchange cartridge was used to extract [^18^F]fluoride from the target water, and physiological saline was used to elute [^18^F]fluoride from the cartridge to the reaction vessel. We used saline instead of a base for the [^18^F]fluoride elution to avoid the need to adjust the pH in the reaction mixture, which improved practicality. In typical cases, the reaction mixture contained 6.8 nmol NOTA-D10CM precursor in sodium acetate buffer at pH 4.0 with acetonitrile as cosolvent. After heating, the product was isolated by HPLC followed by solid phase extraction. The final product was formulated in PBS containing ascorbic acid, in which the ethanol content was less than 10% by volume. It was essential to have ascorbic acid in the formulation to prevent radiolysis. The total radiosynthesis time was 91.8 ± 15.7 minutes (*n* = 10) from the end of the bombardment. The radioactivity yield was 790.0 ± 339.9 MBq (12.9% ± 5.4) with radioactivity concentration of 312.2 ± 140.7 MBq/mL (*n* = 10) starting from 6.1 ± 0.6 GBq of [^18^F]fluoride, and the molar activity was 9.9 ± 4.6 GBq/μmol (*n* = 4) at the EOS. The decay-corrected radiochemical yield was 24.8% ± 13.1. Quality control of the final product was performed with HPLC, and the retention time of Al[^18^F]F-NOTA-D10CM on a C18 reversed-phase column was 6.2 ± 0.4 minutes (Fig. 1b, c).

Al[^18^F]F-NOTA-D10CM was found to be stable over a 4 hour shelf-life test, with consistent radiochemical purity of >99%. The Log*D* value was −3.10, indicating that the tracer was highly hydrophilic, which is in line with the nature of oligosaccharides.

### PET Studies

**Fig. 2:**
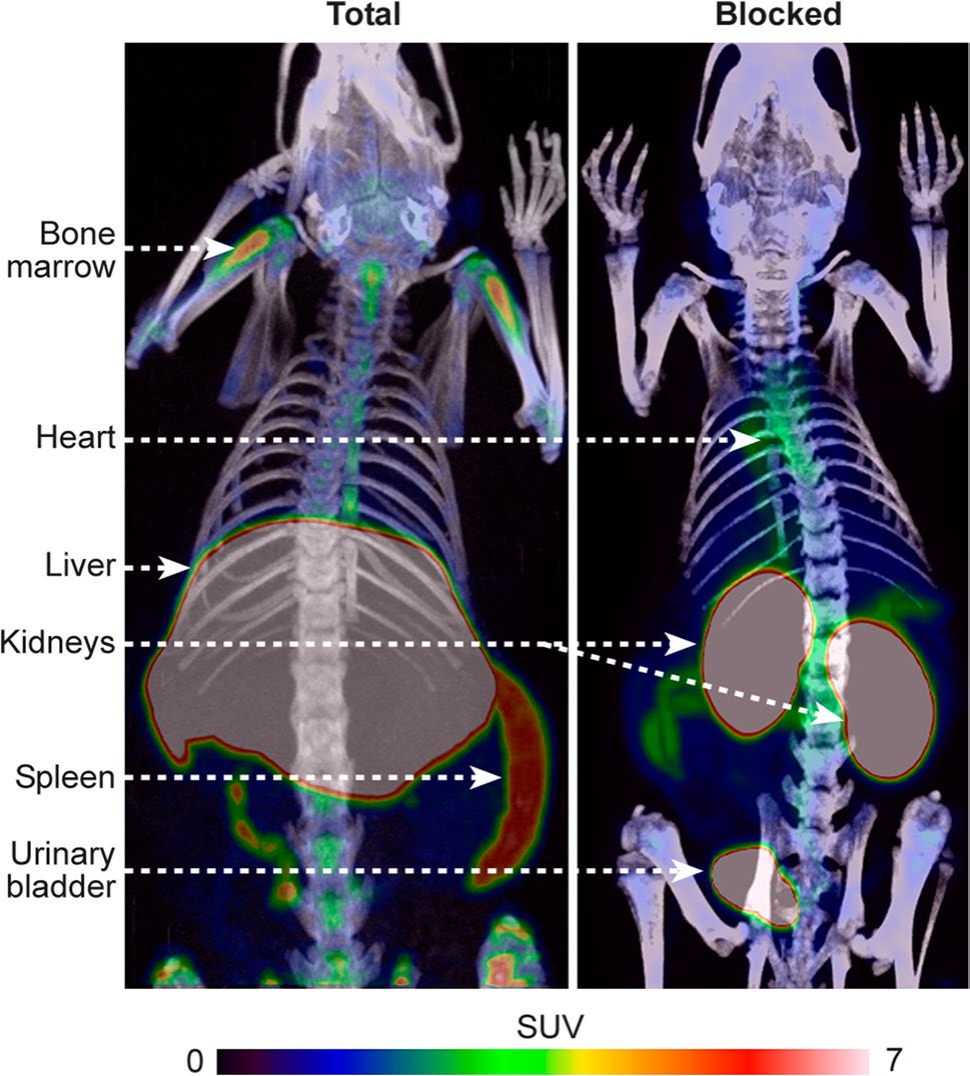
Representative coronal maximum intensity projection Al[^18^F]F-NOTA-D10CM PET/CT images of a healthy rat at 35-60 minutes after injection with (right panel) or without blocking with an excess of mannan (left panel). The blocking effect is especially notable in the liver

**Fig. 3:**
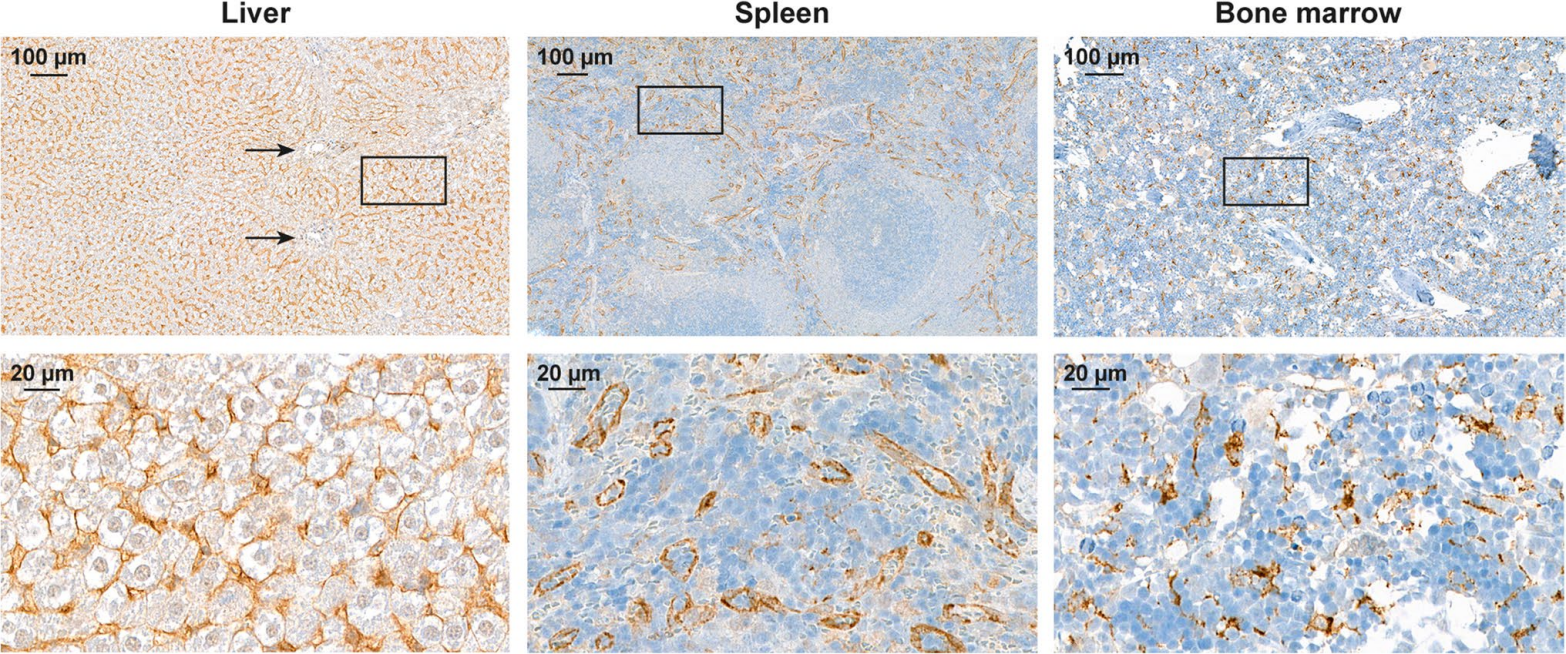
Representative micrographs of liver, spleen and bone marrow from a healthy male Sprague-Dawley rat stained with anti-mannose receptor antibody. Arrows denote liver sinusoidal endothelial cells. Positive immunostaining is shown in brown and nuclei are shown in blue (hematoxylin)

**Fig. 4:**
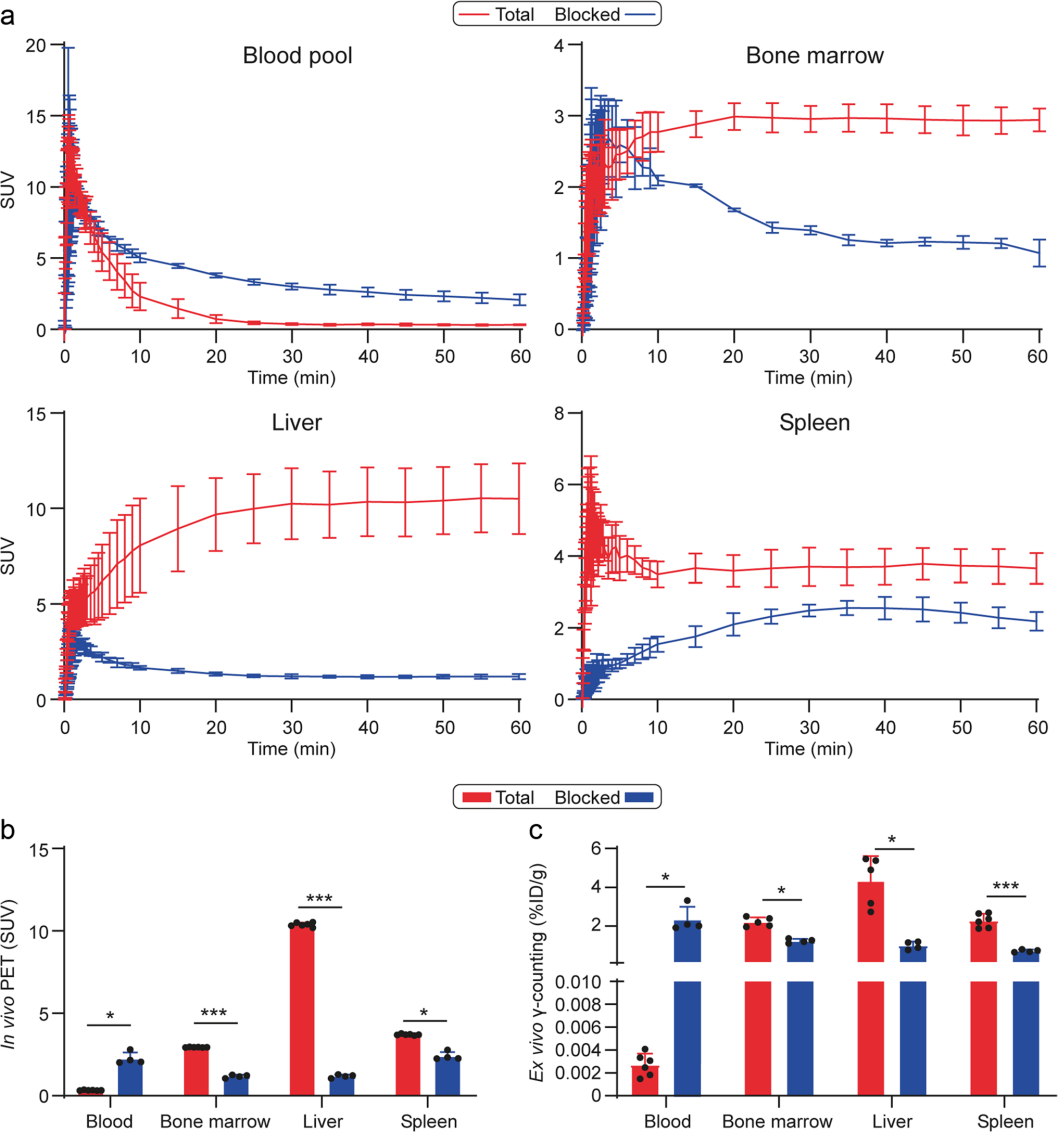
Quantification of total and blocked uptake of Al[^18^F]F-NOTA-D10CM in selected tissues. (**a**) Regional time-activity curves of Al[^18^F]F-NOTA-D10CM. (**b**) Mean standardized uptake value (SUV) on *in vivo* PET imaging at 35-60 minutes postinjection. (**c**) *Ex vivo* gamma counting of excised tissues at 60 minutes postinjection confirms the *in vivo* PET imaging results. Lines represent means, and error bars denote standard deviation (Total *n* = 6, Blocked *n* = 4)

The PET/CT imaging (Fig. 2) showed that the highest SUVs of Al[^18^F]F-NOTA-D10CM occurred in the liver (10.38 ± 0.13), spleen (3.71 ± 0.04), and bone marrow (2.95 ± 0.02) at 35-60 minutes postinjection. These organs are rich in CD206-positive cells, including tissue-resident macrophages, as confirmed by immunohistochemical staining (Fig. 3) [8–11]. The SUVs in salivary glands (1.40 ± 0.02), kidneys (1.37 ± 0.03), and pancreas (1.32 ± 0.05) were low (Supplementary Table 1). The time-activity curves showed that the tracer was cleared from the blood circulation within 20 minutes and reached a plateau in the liver, spleen, bone marrow, and most other tissues (Fig. 4, Supplementary Fig. 2).

To confirm the observations from the *in viv*o imaging, *ex vivo* gamma counting of the excised tissues was carried out. The *ex vivo* results were in line with the *in vivo* PET/CT data, with %ID/g being the highest in liver (4.44 ± 1.32), spleen (2.40 ± 0.45), and bone marrow (1.83 ± 0.95) (Supplementary Table 1).

To confirm the specificity of Al[^18^F]F-NOTA-D10CM uptake, *in vivo* blocking experiments with mannan were performed. Mannan was chosen as a blocking agent on the basis of previous reports describing mannose receptor blockage experiments [12]. According to *in vivo* PET/CT, the blocking resulted in significantly reduced Al[^18^F]F-NOTA-D10CM uptake in CD206-rich tissues: an average reduction of 88.6% in liver, 35.1% in spleen, and 59.9% in bone marrow (Fig. 4, Supplementary Table 1). As expected, the dramatically reduced uptake in the liver, spleen, and bone marrow resulted in prolonged Al[^18^F]F-NOTA-D10CM in the blood circulation and high renal radioactivity in the blocking experiments. However, despite renal excretion, portion of the excreted radioactivity was still observed in the liver. These changes in biodistribution were also clearly visible on whole-body PET/CT images (Fig. 2).

**Fig. 5:**
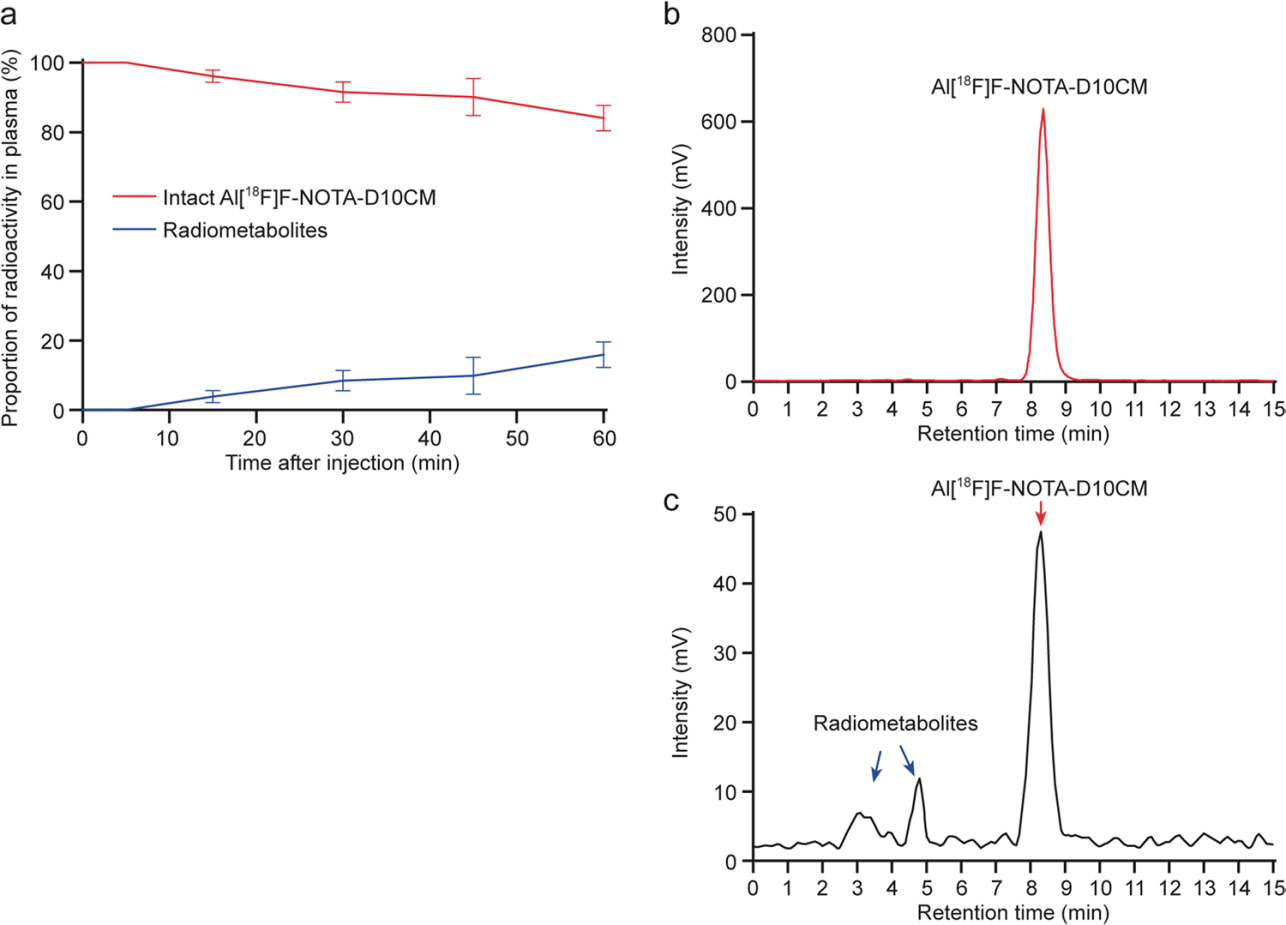
*In vivo* stability of intravenously administered Al[^18^F]F-NOTA-D10CM in rats. (**a**) Percentage of intact tracer from total plasma radioactivity at different time points after injection. Lines represent means, and error bars denote standard deviation (*n* = 3-4 for each time point). (**b**) Representative radio-HPLC chromatograms of intact Al[^18^F]F-NOTA-D10CM and (**c**) rat plasma at 60 minutes postinjection

The *in vivo* stability of Al[^18^F]F-NOTA-D10CM was studied in rats by taking serial blood samples up to 60 minutes after injection followed by HPLC analysis of protein-free plasma under radioactivity detection. The intact tracer accounted for 100% ± 0, 96.2% ± 1.8%, 91.6% ± 3.0%, 90.1% ± 5.3%, and 84.1% ± 3.7% of total plasma radioactivity at 5, 15, 30, 45, and 60 minutes after injection, respectively, indicating excellent *in vivo* stability (Fig. 5). We also measured the distribution of radioactivity among the different components of the blood: on average, 7.2% ± 4.0% of the blood radioactivity was found in cells and 92.8% ± 4.0% in plasma, while 66.3% ± 6.5% of plasma radioactivity was free-form, and 33.7% ± 6.5% was bound to proteins.

## Discussion

In this work, we describe the Al[^18^F]F-labeling of NOTA-conjugated mannosylated dextran derivative and demonstrate its accumulation in mannose receptor-rich tissues by *in vivo* PET imaging. To our knowledge, this is the first time that the mannosylated dextran has been labeled with Al[^18^F]F-labeling technique. Accordingly, facile incorporation of ^18^F into NOTA-D10CM and promising first PET studies encourage further studies.

In a previous study by Pirmettis and coworkers, dextran (molecular weight 11.8 kDa) was used as a backbone, to which a mannose moiety for targeting and a cysteine moiety for chelation of ^99m^Tc were attached resulting DCM20 with 24 units of mannose per mol of dextran (22.3 kDa) [3]. The ^99m^Tc-labeled DCM20 was intended for CD206-targeted sentinel lymph node mapping by subcutaneous or intradermal injection. Further studies by Papasavva and coworkers compared a series of different size mannosylated dextrans (21.3-805.6 kDa) with 19-645 units of mannose. Their results revealed that ^99m^Tc-labeled D75CM with 74 mannose units (111.2 kDa) was superior because of the highest sentinel lymph node accumulation via subcutaneous injection and accumulation in the liver after intravenous administration were observed [5]. Due to excellent imaging performance and favorable biokinetics, we kept the molecule structure as similar as possible in this study. Accordingly, we did not omit the cysteine moiety, even though it is not needed for the purpose of ^18^F-labeling in the current situation. In this study, we aimed to reveal the potential of smaller size D10CM (21.3 kDa) with 17 mannose units for mannose receptor targeting by PET imaging modality using an intravenous route for whole body distribution of the Al[^18^F]F-NOTA-D10CM.

The precursor compound NOTA-D10CM was conveniently synthesized by *N-*acylation of the amino group of the cysteine residues under slightly alkaline conditions, and purified by ultrafiltration. The easy availability of the precursor facilitates its potential use in nuclear medicine, including PET imaging. Among the available ^18^F-fluorination methods, Al[^18^F]F-labeling is one of the most straightforward techniques. Al[^18^F]F-labeling is based on the tight affinity of fluoride to aluminum [13]. To fix Al[^18^F]F into a radiopharmaceutical, several good chelators, including NOTA, restrained complexing agent (RESCA), 1,4,7-triazacyclononane-1,4-diacetate (NODA), and 1,4,7-triazacyclononane,1-glutaric acid-4,7-acetic acid (NODAGA) have been developed [14]. Among them, NOTA is probably the most often used chelator in the clinical setting. The Al[^18^F]F-NOTA complex has shown excellent stability *in vivo*, not only in animals but also in humans [15, 16]. To our knowledge, there are no clinical safety issues associated with the moiety of Al[^18^F]F-NOTA in the radiopharmaceutical used for imaging purposes. Therefore, we decided to use Al[^18^F]F-NOTA chelation chemistry for the labeling of D10CM (Fig. 1). Indeed, the radiosynthesis protocol was rather reproducible and robust. Although the decay-corrected radiochemical yield of ~25% was only moderate, the radioactivity yield of ~800 MBq from a starting activity of 6 GBq of [^18^F]fluoride was adequate for preclinical PET applications.

We noticed that it was important to store the 0.1 M acetate buffer in small aliquots, because frequent opening the vial caused change in pH due to evaporation, which contributed to the failure of the radiosynthesis. We also observed that a high concentration (>50%) of organic solvents (e.g., ethanol and acetonitrile) caused precipitation of Al[^18^F]F-NOTA-D10CM. Therefore, we kept the concentration of organic solvents below 40% in chromatographic experiments, reaction mixtures, and solid phase extraction. For example, in the step involving elution of Al[^18^F]F-NOTA-D10CM from the solid phase extraction tC18 cartridge to the end product vial, it was critical to use 30% ethanol instead of the 50-100% that we usually use for other radiopharmaceuticals.

Mannosylated dextran derivatives have also been successfully ^18^F-labeled by covalent bond formation. For example, Ting and coworkers have applied boron-based ^18^F-fluorination method to prepare a [^18^F]BOMB Lymphoseek with the molar activity of up to 1.85 GBq/μmol [17]. For comparison, the molar activity of our Al[^18^F]F-NOTA-D10CM was 10 GBq/μmol, which is moderate.

Ultimately, the Al[^18^F]F-NOTA-D10CM is intended for imaging of mannose receptor (CD206) expression. However, as a first step, we performed PET studies in healthy rats to evaluate its biodistribution and *in vivo* stability. Indeed, our results revealed that vast majority of Al[^18^F]F-NOTA-D10CM had cleared from blood circulation within 20 minutes and accumulated in liver, spleen and bone marrow rich in mannose receptor-positive cells (Fig. 3 and 4, Supplementary Table 1). Furthermore, we found that the *in vivo* stability of Al[^18^F]F-NOTA-D10CM was excellent, with approximately 84% of total plasma radioactivity originating from intact Al[^18^F]F-NOTA-D10CM at 60 minutes after i.v. injection (Fig. 5). Interestingly, our results are in line with the study by Choi and coworkers, in which intravenously administered ^68^Ga-labeled mannosylated human serum albumin showed a similar biodistribution trend especially in the liver, spleen and bone marrow [18], i.e. organs associated with the reticuloendothelial system. To confirm the specificity of Al[^18^F]F-NOTA-D10CM uptake, an *in vivo* blocking study with molar excess of mannan was performed. Indeed, the uptake in liver, spleen and bone marrow was significantly reduced. The blocking effect was clearly seen not only in the *ex vivo* tissue radioactivity measurements, but also in *in vivo* PET images. When the uptake is blocked in the mannose receptor-rich organs, increased amount of Al[^18^F]F-NOTA-D10CM becomes available in the blood circulation and increased uptake into other tissues, including muscle, is observed. To evaluate the *in vivo* stability of Al[^18^F]F-NOTA-D10CM in rats, blood samples were taken at five time points within one hour and protein-free plasma was isolated for metabolite analysis by HPLC. In order to precipitate plasma proteins, 10% sulfosalicylic acid had to be used instead of the commonly used organic solvent acetonitrile. This was to avoid precipitation of Al[^18^F]F-NOTA-D10CM with a high concentration of organic solvents in the sample matrix, as described in above.

## Conclusion

To conclude, the chelator-conjugated mannosylated dextran derivative NOTA-D10CM was synthesized to achieve efficient and straightforward labeling with Al[^18^F]F chelation chemistry. The new radiopharmaceutical, Al[^18^F]F-NOTA-D10CM, was produced with a high quality and moderate yield, and presented excellent *in vitro* and *in vivo* stability in rats. Al[^18^F]F-NOTA-D10CM was rapidly cleared from the blood circulation and showed the highest uptake in the macrophage-rich organs of liver, spleen, and bone marrow. *In vivo* blocking experiments established that the uptake is specific and in line with high mannose receptor expression. On the basis of these favorable characteristics, we consider that further translational studies in animal disease models are warranted.

### Supplementary Information

ESM 1: The online version contains supplementary material. (DOCX 2.76 MB)

## Abbreviations

^18^F: fluorine-18
^99m^Tc: technetium-99m
%ID/g: percentage of the injected radioactivity dose per gram of tissue weight
Al[^18^F]F: aluminum fluoride-18
*c*: concentration
CD206: cluster of differentiation 206
CT: computed tomography
D10CM: dextran derivative containing cysteine and mannose moieties
HPLC: high-performance liquid chromatography
NMR: nuclear magnetic resonance
NOTA: 1,4,7-triazacyclononane-1,4,7-triacetic acid
NOTA-NHS: *N*-hydroxysuccinimide-activated ester of NOTA
PBS: phosphate-buffered saline
PET: positron emission tomography
ROI: region of interest
SD: standard deviation
SUV: standardized uptake value
TFA: trifluoroacetic acid; UV: ultra-violet
