# Supplementary material for: Aluminum Fluoride-18 Labeled Mannosylated Dextran: Radiosynthesis and Initial Preclinical Positron Emission Tomography Studies
Source: Mol Imaging Biol. 2023 Apr 4;25(6):1094–103. doi: 10.1007/s11307-023-01816-7 (PMC10728250; doi:10.1007/s11307-023-01816-7)
Supplement: Supplementary file 1 — The online version contains supplementary material. (DOCX 2.76 MB) [file 11307_2023_1816_MOESM1_ESM.docx]

Supplementary Information to:

**Aluminum fluoride-18 Labeled Mannosylated Dextran: Radiosynthesis and Initial Preclinical Positron Emission Tomography Studies**

Putri Andriana^1^, Konstantina Makrypidi^2^, Heidi Liljenbäck^1,3^, Johan Rajander^4^, Antti Saraste^1,5,6^, Ioannis Pirmettis^2^, Anne Roivainen^1,3,5,7*^, Xiang-Guo Li^1,7,8*^

^1^Turku PET Centre, University of Turku, FI-20520 Turku, Finland

^2^Institute of Nuclear and Radiological Science and Technology, Energy and Safety, NCSR “Demokritos”, 15310 Athens, Greece

^3^Turku Center of Disease Modeling, University of Turku, FI-20520 Turku, Finland

^4^Accelerator Laboratory, Åbo Akademi University, FI-20520 Turku, Finland

^5^Turku PET Centre, Turku University Hospital, FI-20520 Turku, Finland

^6^Heart Center, Turku University Hospital and University of Turku, FI-20520 Turku, Finland

^7^InFLAMES Research Flagship Center, University of Turku, FI-20520 Turku, Finland

^8^Department of Chemistry, University of Turku, FI-20014 Turku, Finland

***Corresponding Authors:** Assistant Professor Xiang-Guo Li, PhD, Turku PET Centre, Kiinamyllynkatu 4-8, FI-20520 Turku, Finland; Phone: +358504485069; E-mail: [xiali@utu.fi](mailto:xiali@utu.fi)

Professor Anne Roivainen, PhD, Turku PET Centre, Kiinamyllynkatu 4-8, FI-20520 Turku, Finland; Phone: +35823132862; E-mail: [anne.roivainen@utu.fi](mailto:anne.roivainen@utu.fi)

*Equal contribution

**First Author:** Putri Andriana, PhD student, Turku PET Centre, Kiinamyllynkatu 4-8, FI-20520 Turku, Finland; Phone: +358449449440; E-mail: [putri.p.andriana@utu.fi](mailto:putri.p.andriana@utu.fi)

**
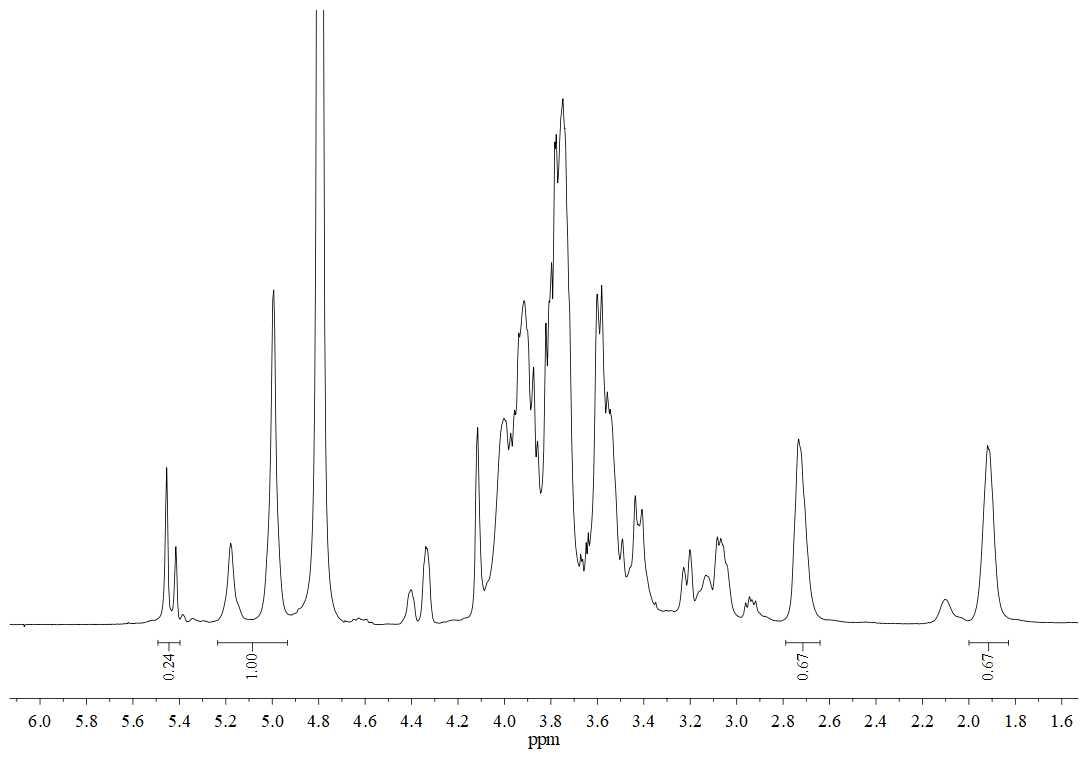
**

**Supplementary Fig. 1** Nuclear magnetic resonance (NMR) data of NOTA-D10CM. NMR data were acquired in D_2_O at 25°C on a 500-MHz NMR Bruker DRX Avance spectrometer. D10CM was prepared according to previous methods [1−3] and conjugated with NOTA-NHS as described in the manuscript. On the basis of the NMR data, DCM contained 7 free and 17 mannosylated S-derivatized cysteines. In the structure of NOTA-D10CM, there was at least one NOTA chelator per D10CM molecule.

**
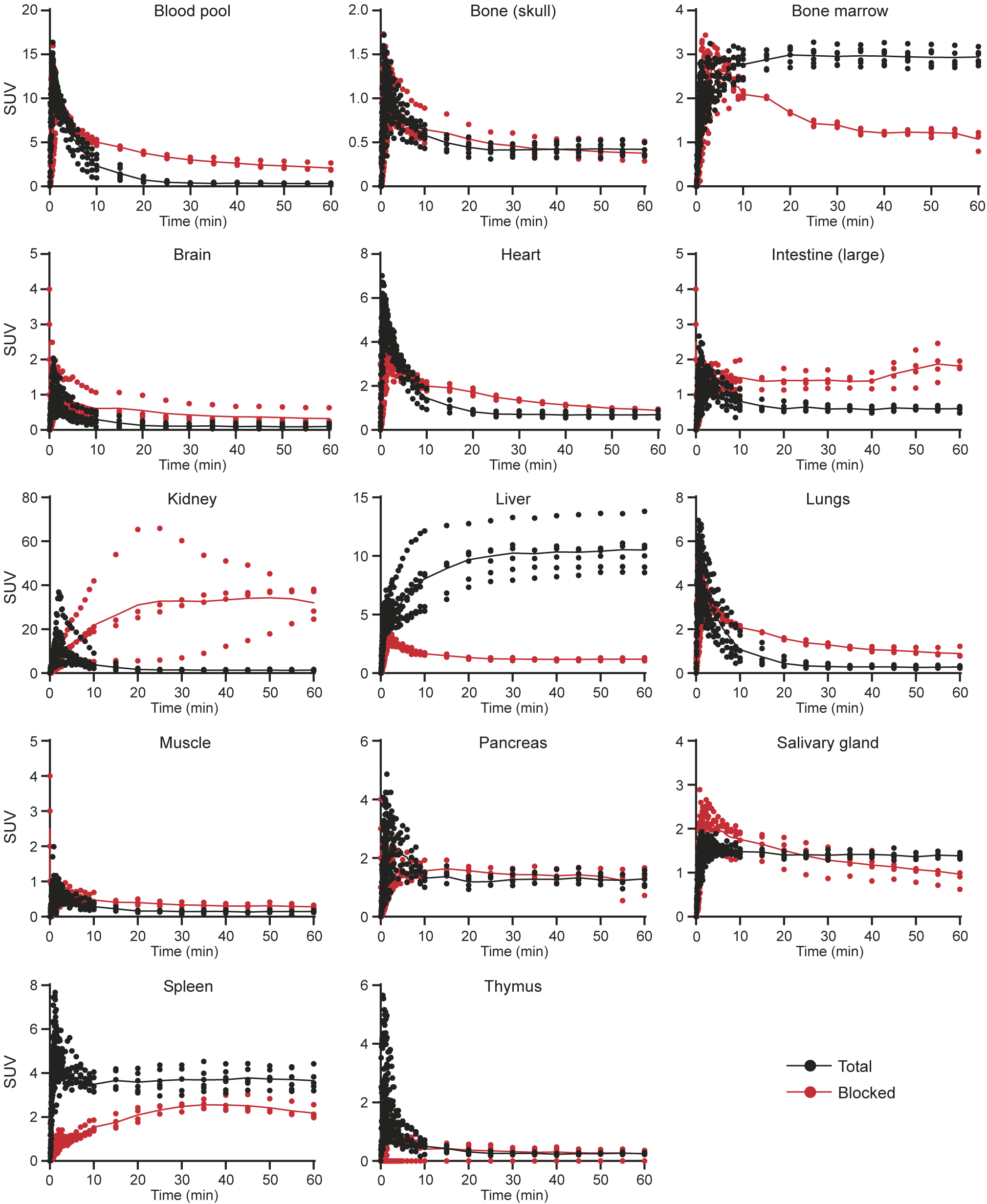
**

**Supplementary Fig. 2** Time-activity curves of total and blocked uptake of Al[^18^F]F-NOTA-D10CM in various rat organs. Each circle represents a single rat, and the lines are the mean standardized uptake value (SUV).

**Supplementary Table 1.** Biodistribution of intravenously injected Al[^18^F]F-NOTA-D10CM in healthy rats

| Tissue | *In vivo* PET  (SUV_mean_, 35−60 minutes postinjection) | | *Ex vivo* gamma counting  (%ID/g, 60 minutes postinjection) | |
| --- | --- | --- | --- | --- |
|  | Total  (*n* = 6) | Blocked  (*n* = 4) | Total  (*n* = 6) | Blocked  (*n* = 4) |
| Blood | 0.33 ± 0.02* | 2.26 ± 0.37 | 0.00 ± 0.00* | 2.33 ± 0.66 |
| Bone | 0.42 ± 0.06 | 0.40 ± 0.09 | 0.14 ± 0.02 | 0.25 ± 0.11 |
| Bone marrow | 2.95 ± 0.02*** | 1.18 ± 0.09 | 2.21 ± 0.23** | 1.23 ± 0.11 |
| Brain | 0.10 ± 0.01 | 0.34 ± 0.20 | 0.00 ± 0.00* | 0.05 ± 0.02 |
| Heart | 0.61 ± 0.14** | 0.98 ± 0.03 | 0.14 ±0.11* | 0.64 ± 0.14 |
| Kidney | 1.37 ± 0.03* | 38.09 ± 1.75 | 0.47 ± 0.10* | 57.38 ± 22.75 |
| Intestine, large | 0.60 ± 0.02* | 1.71 ± 0.23 | 0.24 ± 0.02* | 0.61 ± 0.12 |
| Intestine, small | ND | ND | 0.26 ± 0.08 | 0.47 ± 0.16 |
| Liver | 10.38 ± 0.13*** | 1.19 ± 0.10 | 4.33 ± 1.28* | 0.99 ± 0.20 |
| Lung | 0.28 ± 0.01* | 0.96 ± 0.23 | 0.13 ± 0.01* | 0.84 ± 0.29 |
| Muscle | 0.14 ± 0.01* | 0.29 ± 0.05 | 0.02 ± 0.00* | 0.13 ± 0.04 |
| Pancreas | 1.32 ± 0.05 | 1.34 ± 0.29 | 0.40 ± 0.08* | 0.70 ± 0.15 |
| Salivary gland | 1.40 ± 0.02 | 1.05 ± 0.26 | ND | ND |
| Spleen | 3.71 ± 0.04* | 2.41 ± 0.25 | 2.28 ± 0.36*** | 0.71 ± 0.07 |
| Testis | ND | ND | 0.04 ± 0.01* | 0.12 ± 0.03 |
| Thymus | 0.25 ± 0.01 | 0.27 ± 0.18 | 0.04 ± 0.01* | 0.09 ± 0.03 |

**P* < 0.05, ***P* < 0.001, ****P* < 0.0001 *vs.* Blocked. ND, not determined.

**References**

1. Pirmettis I, Arano Y, Tsotakos T, et al. (2012) New ^99m^Tc(CO)_3_ mannosylated dextran bearing S-derivatized cysteine chelator for sentinel lymph node detection. Mol Pharm 9:1681–1692.
2. Papasavva A, Shegani A, Kiritsis C, et al. (2021) Comparative study of a series of ^99m^Tc(CO)_3_ mannosylated dextran derivatives for sentinel lymph node detection. Molecules 26:4797.
3. Tsoukalas C, Lazopoulos, A, Boschetti, F, et al. (2014) Labeling of a NOTA mannosylated dextran with ^68^Ga. Nucl Med Biol 41:801.
